# A painful nodule on the right great toe

**DOI:** 10.1016/j.jdcr.2024.06.040

**Published:** 2024-07-22

**Authors:** Josiah A. Williams, Erica Ghareeb, Roxann Powers, Grant McChesney, Ralph Condon Hughes, Colleen Beatty

**Affiliations:** a Department of Dermatology, West Virginia University School of Medicine, Morgantown, West Virginia; b Department of Orthopedic Surgery, West Virginia University School of Medicine, Morgantown, West Virginia

**Keywords:** dermatology, digital papillary adenocarcinoma, medical dermatology, oncology, surgical dermatology

## History

**Fig 1:**
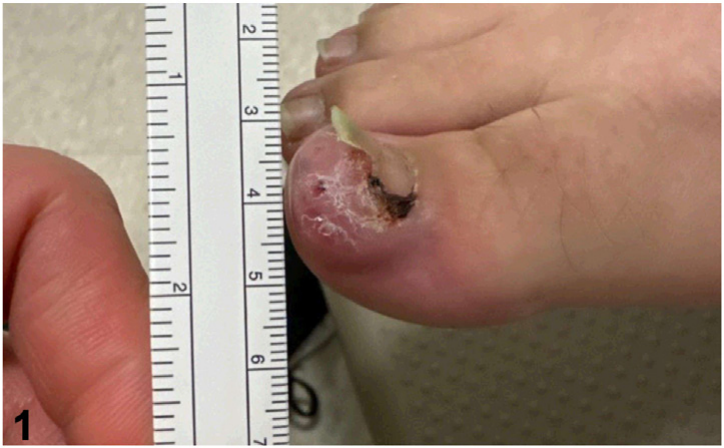

**Fig 2:**
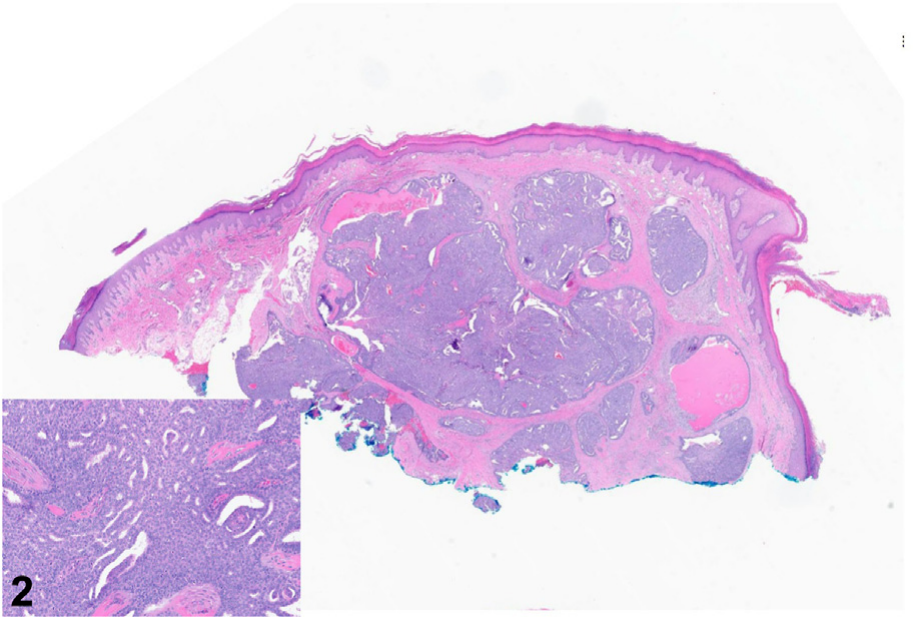

A 35-year-old woman with no relevant medical history presented to the dermatology clinic for a lesion on her right great toe present for 1 year. The patient reported that she first noticed a faint brown-to-black discoloration under the skin of the distal toe that progressively enlarged. It had become tender over the preceding months. Exam revealed a 1.8 cm dark purple nodule on the distal right great toe extending beneath the nail (Fig 1). A shave biopsy was performed (Fig 2).

**Question 1: What is the patient’s diagnosis?**

A. Acral lentiginous melanoma
B. Pyogenic granuloma
C. Digital papillary adenocarcinoma
D. Squamous cell carcinoma
E. Atypical mycobacterial infection

**Answers:**

A. Acral lentiginous melanoma – Incorrect. Acral lentiginous melanomas may present as a rapidly enlarging dark papule on acral surfaces, including the toes.^1^ Although this was included on the differential diagnosis, histopathology would reveal an invasive proliferation of atypical melanocytes, unlike the case described.^1^
B. Pyogenic granuloma – Incorrect. Pyogenic granulomas may present on digits, but histopathology would reveal a well-circumscribed proliferation of capillaries arranged in a lobular configuration within the dermis.^1^ Epithelial collarettes are often seen on lateral margins, which result from peripheral adnexal hyperplasia or downward growth of rete ridges.^1^
C. Digital papillary adenocarcinoma – Correct. Digital papillary adenocarcinoma (DPAC) presents as a growing nodule on a digit with histopathology findings of a dermal-based adnexal tumor with cytologic atypia, solid and cystic spaces, and papillary projections, with scattered mitotic figures.^2^ Although the typical presentation is on the finger of an elderly man, they may present on toes and in younger patients and are helpful to include in the differential diagnosis of digital lesions.^2^
D. Squamous cell carcinoma – Incorrect. Squamous cell carcinoma may present on digits, although they are characteristically erythematous and scaly, sometimes with ulceration.^1^ Furthermore, pathology would typically show invasive collections of eosinophilic keratinocytes arising from the epidermis, though cell morphology may vary for poorly differentiated squamous cell carcinoma.^1^
E. Atypical mycobacterial infection – Incorrect. Atypical mycobacterial infection, such as *Mycobacterium marinum*, may present as a painful nodule which may be solitary or exhibit a lymphocutaneous “sporotrichoid” pattern.^1^ Unlike this case, biopsy results would typically show granulomatous inflammation with mycobacterial organisms on acid-fast bacteria staining.^1^

**Question 2: Which of the following immunohistochemistry (IHC) or *in situ* hybridization (ISH) studies would be most appropriate to confirm the diagnosis?**

A. CD34 IHC
B. Human papillomavirus 16 (HPV16) ISH
C. CK20 IHC
D. Pleckstrin homology like domain family A member 1IHC
E. HPV42 ISH

**Answers:**

A. CD34 IHC – Incorrect. CD34 is an endothelial marker as well as a marker of hair outer root sheath differentiation; it may be positive in various neoplasms, including vascular neoplasms and dermatofibrosarcoma protuberans (DFSP).^1^ CD34 can be a helpful stain for identifying CD34-positive fibroblasts in fibrous and fibrohistiocytic proliferations as well.^1^
B. Human papillomavirus 16 (HPV16) ISH – Incorrect. human papillomavirus 16 is an oncogenic strain of HPV typically associated with squamous cell carcinoma, especially cervical and penile squamous cell carcinoma.^1^ It is the most frequent strain identified in HPV-associated digital squamous cell carcinomas, but other strains may be implicated, including HPV-31 and HPV-33.^1^
C. CK20 IHC – Incorrect. CK20 stains positive in various carcinomas.^1^ CK20 stains positive in Merkel cell carcinoma with a characteristic perinuclear globule pattern.^1^ Clinical and histopathologic presentation are not consistent with these entities.
D. Pleckstrin homology like domain family A member 1IHC – Incorrect. Pleckstrin homology like domain family A member 1 is a hair follicle bulge marker positive in trichoblastomas, trichoepitheliomas, and desmoplastic trichoepitheliomas.^1^ These lesions do not typically present on digits.^1^
E. HPV42 ISH – Correct. HPV42 was previously thought to be a nononcogenic subtype of HPV; however, it is now implicated in digital papillary adenocarcinoma.^3^ Approximately 96% of digital papillary adenocarcinomas will be positive for HPV42 via in situ hybridization.^3^ This recent advancement in our understanding of the pathogenesis of this tumor allows for distinction of well-differentiated DPAC from other benign adnexal tumors. Most oncogenic HPV subtypes mediate oncogenesis primarily by “early” proteins, especially E6 and E7.^3^ E6 protein functions by ubiquitinating p53 for proteasomal degradation, while E7 disrupts retinoblastoma (Rb) protein.^3^ Unlike most oncogenic HPV subtypes, HPV42 E6 protein does not degrade p53, and it mediates oncogenicity primarily via its E7 protein.^3^

**Question 3: Which of the following is the most appropriate next step in management?**

A. Wide local excision in dermatology clinic
B. Referral to Mohs micrographic surgery for removal of the primary lesion without any staging evaluation
C. Referral to orthopedic surgical oncology for staging and wide resection with possible amputation
D. Referral to oncology for radiation and chemotherapy without surgical intervention
E. Close monitoring in clinic as most cases involute

**Answers:**

A. Wide local excision in dermatology clinic – Incorrect. Wide local excision could be considered for DPAC, but it may not be feasible given the digital location.^4^ Furthermore, this would not be an appropriate procedure to perform in dermatology clinic since deeper tissues, including tendon and bone, would be involved. Lastly, staging would be warranted in addition to surgical management.
B. Referral to Mohs micrographic surgery for removal of the primary lesion without any staging evaluation – Incorrect. Mohs micrographic surgery may be utilized successfully in the treatment of DPAC.^5^ However, this is only performed in conjunction with appropriate investigation of metastases, such as sentinel lymph node biopsy or imaging studies.^5^
C. Referral to orthopedic surgical oncology for staging and wide resection with possible amputation – Correct. Definitive surgical removal of the primary lesion plus investigation of metastatic disease is recommended for DPAC, which could include staging scans plus or minus sentinel lymph node biopsy.^4^ Up to 41% of DPAC cases may have metastatic disease, though recent case series suggest the percentage to be smaller.^4^
D. Referral to oncology for radiation and chemotherapy without surgical intervention – Incorrect. The role of radiation and chemotherapy in the treatment of DPAC has not been well characterized, and removal of the primary lesion and staging would be needed before these options were considered.^4^
E. Close monitoring in clinic as most cases involute – Incorrect. Close monitoring is not appropriate management for DPAC given the aggressive nature of this cancer and potential for metastasis.^4^

## Conflicts of interest

None disclosed.
